# Sex, Race, and Age Differences in Observed Years of Life, Healthy Life, and Able Life among Older Adults in The Cardiovascular Health Study

**DOI:** 10.3390/jpm5040440

**Published:** 2015-11-25

**Authors:** Stephen M. Thielke, Paula H. Diehr, Laura M. Yee, Alice M. Arnold, Ana R. Quiñones, Heather E. Whitson, Mini E. Jacob, Anne B. Newman

**Affiliations:** 1 Department of Psychiatry and Behavioral Sciences, University of Washington, Seattle, WA 98195, USA; 2 Geriatric Research, Education, and Clinical Center, Puget Sound Veterans Affairs Medical Center, Seattle, WA 98108, USA; 3 Department of Biostatistics, University of Washington, Seattle, WA 98195, USA; E-Mails: pdiehr@u.washington.edu (P.H.D.); laurayee@u.washington.edu (L.M.Y.); arnolda@u.washington.edu (A.M.A.); 4 Department of Health Services, University of Washington, Seattle, WA 98195, USA; 5 Department of Public Health and Preventive Medicine, Oregon Health and Science University, Portland, OR 97239, USA; E-Mail: quinones@ohsu.edu; 6 Department of Medicine (Geriatrics) and the Aging Center, Duke University Medical Center, Durham, NC 27708, USA; E-Mail: heather.whitson@duke.edu; 7 Geriatric Research, Education, and Clinical Center, Durham VA Medical Center, Durham, NC 27705, USA; 8 Department of Epidemiology, University of Pittsburgh, Pittsburgh, PA 15260, USA; E-Mails: MEJ49@pitt.edu (M.E.J.); NewmanA@edc.pitt.edu (A.B.N.)

**Keywords:** able life, active life, healthy life, longevity, ADL

## Abstract

*Objective*: Longevity fails to account for health and functional status during aging. We sought to quantify differences in years of total life, years of healthy life, and years of able life among groups defined by age, sex, and race. *Design*: Primary analysis of a cohort study. *Setting*: 18 years of annual evaluations in four U.S. communities. *Participants*: 5888 men and women aged 65 and older. *Measurements*: Years of life were calculated as the time from enrollment to death or 18 years. Years of total, healthy, and able life were determined from self-report during annual or semi-annual contacts. Cumulative years were summed across each of the age and sex groups. *Results*: White women had the best outcomes for all three measures, followed by white men, non-white women, and non-white men. For example, at the mean age of 73, a white female participant could expect 12.9 years of life, 8.9 of healthy life and 9.5 of able life, while a non-white female could expect 12.6, 7.0, and 8.0 years, respectively. A white male could expect 11.2, 8.1, and 8.9 years of life, healthy life, and able life, and a non-white male 10.3, 6.2, and 7.9 years. Regardless of starting age, individuals of the same race and sex groups spent similar amounts (not proportions) of time in an unhealthy or unable state. *Conclusion*: Gender had a greater effect on longevity than did race, but race had a greater effect on years spent healthy or able. The mean number of years spent in an unable or sick state was surprisingly independent of the lifespan.

## 1. Introduction

Over the past 30 years, research on aging has emphasized health outcomes that capture more than longevity. In particular, investigators have distinguished between amount of time spent *alive*, the amount of time *healthy*, and the amount of time *able to perform activities of daily living without difficulty*. These characterize different dimensions of the aging process, since individuals may vary in their health or ability status even if living the same number of years. Instead of using a single metric to embody important differences between groups, there is merit in looking separately at lifespan, health, and functioning.

Studies estimating able or healthy life expectancy have found that there are substantial differences between lifespan and the amount of time spent in a state of health (healthy life expectancy) or without any functional impairments (able life expectancy). Focusing only on longevity fails to fully describe inequalities between sexes, races, and geographical areas in able and healthy life expectancy [1,2,3]. There are mixed results about the interacting effects of age, gender, and race on able life expectancy. For instance, one analysis found that women had a higher able life expectancy than men at age 65, and that men had a higher able life expectancy than women at age 85 [4], but other work found no significant differences between men and women [5]. The latter study found that black older adults had lower able life expectancy, but in another study black men and women aged 75 years or older had higher able life expectancy than whites [6].

Previous research has not fully addressed the differences in the time spent in various states of health and functional ability. Almost all previous research has used life tables, increment-decrement, or Markov panels—estimates of health states or transition probabilities at specific ages—rather than observed data [3,5,6,7,8,9,10,11]. These analyses are based on many assumptions, and also require extensive amounts of data for all subgroups in order to establish for reliable estimates. Therefore analyses with fewer assumptions are warranted [12]. The age ranges examined have been mainly truncated at 75 or 80, giving an incomplete perspective on aging. Analyses that measure rather than estimate years of healthy or able life among large samples are needed in order to ascertain the interactions between age, sex, and gender on future health.

Measurements of the health status of different populations of older adults have relevance for the epidemiology of aging, public health, research on racial and ethnic and gender disparities, and intervention planning. We sought to quantify observed differences among groups defined by age, sex, and race in years of life, years of healthy life (defined by self-rated health), and years of able life defined by the absence of any impairments in Activities of Daily Living (ADLs), in a large group of older Americans in the Cardiovascular Health Study who were observed for 18 years. Unlike previous approaches, we used annual observations to compute the total amount of years of able, healthy, and total life for each person. We hypothesized that years of able and years of healthy life would differ based on age, sex, and race, and that years of able life, years of healthy life, and years of total life would show similar trends.

## 2. Methods

### 2.1. Sample

The Cardiovascular Health Study (CHS) is an ongoing observational cohort study of American older adults, initiated to ascertain risk factors for cardiovascular disease in older adults. In 1989 and 1990, 5201 participants (Cohort 1) were enrolled from Medicare eligibility lists in four communities: Sacramento County, California; Allegheny County, Pennsylvania, Forsyth County, North Carolina, and Washington County, Maryland. Participants were excluded if they were institutionalized, not able to ambulate in their home, not able to be interviewed, were receiving hospice care or radiation or chemotherapy for cancer, or did not expect to remain in the area for at least three years. Because minority enrollment was initially low, in 1992 and 1993 an additional 687 African-American participants were recruited (Cohort 2), from the same communities except for the Maryland site, which had few potential participants. Participants received annual in-person interviews and 6-month telephone calls until 1999, and thereafter received telephone calls twice per year. We included data for the first 18 years of follow-up from both cohorts (through 2008 for Cohort 1 and through 2011 for Cohort 2). Details of the study design and sampling methods have been described elsewhere [13]. The institutional review board (IRB) of each of the sites and the Coordinating Center approved the study.

### 2.2. Sociodemographic Categories

We considered 5-year age groups up to age 80, and a final group of age ≥80 years. For this analysis, we examined the self-reported race categories of white and non-white. Few participants (*n* = 39) reported a race other than black or white; they were categorized as non-white.

### 2.3. Annual Ascertainment of Status

Vital status was ascertained every 6 months by phone contact or by notification from a proxy or family member, review of local obituaries, or from Center for Medicare Studies records. Date of death, as confirmed by death certificate, medical records, telephone interview with a proxy respondent, and/or Center for Medicare Services records, was used to calculate total years of life (YOL), from a total of 18.

Years of healthy life (YHL) were measured semi-annually using a question about self-rated health, “How would you rate your health in general?”, with the response categories Excellent, Very Good, Good, Fair, or Poor. This measure has become well-established as a general and highly predictive gauge of health status [14]. Using an established procedure [15], years in which the participant responded Excellent, Very Good, or Good were categorized as healthy, and the remainder (Fair or Poor) were categorized as not healthy. The total number of years in a healthy state, from a total of 18, were calculated as described below.

Years of able life (YAL) were measured by questions about activities of daily living (ADL) in six domains: walking around the home, getting out of bed, eating, dressing, bathing, and using the toilet. Participants were asked if they had any difficulty with each of these activities. If they reported no difficulty for all of them, they were considered to be able for that year; otherwise there were considered to be not able. Each participant’s total years with able status were summed. Data were collected every year for 9 years, and every other year thereafter.

### 2.4. Standardization of Health Scales

Missing data were imputed by linear interpolation from the closest observations before and after the missing data [16]. Overall, 45% of all observations involved such an intervening missing value. A small number of missing observations without any previous (or without any subsequent) observations were imputed by the first observation carried backward (0.03% of observations), or the last observation carried forwards (0.9% of observations). For the purposes of imputation, the self-rated health question was first recalibrated on a 100-point scale, based on the probability of reporting excellent, very good, or good self-rated health the following year [17]. ADL difficulties were coded in a similar way [18]. To calculate the number of years of healthy or able life between baseline and the 18th measure in which the person was healthy or able, we estimated the area under the curve, using the trapezoidal method [19]. Details of the standardization and imputation methods have been published previously [18].

### 2.5. Special Methods for ADL

Activities of daily living were measured annually from the start of the study until 1999 using the same measure, but from 2000 to 2004 they were assessed using a different question about change in functioning during the previous year, which could not be mapped directly onto the earlier measures. Starting in 2005, all the measurements reverted back to the earlier question, and were obtained semi-annually. For the intervening time period, all the observations about YAL were set to missing and then imputed as described above. We then applied a post-adjustment to this data, by reweighting the imputed values to ensure that the averages during the five-year period were consistent with the average observed values in the surrounding years (1999 and 2005) [18]. Analyses: Bar graphs were used to illustrate the YOL, YHL and YAL totals by age, sex and race groups. Because death is a possible state, these graphs were adjusted to sum to the same total (18 years). To illustrate the variability in our estimates, we also created error bar graphs for the mean YOL, YHL, and YAL for each age and sex group. Linear regression was used to estimate the effect of age, sex and race on the outcomes, with age modeled linearly, after confirming the approximate linearity of the relationships with linear splines. We analyzed the data and generated figures using R 2.13.0 (R Development Core Team, Vienna, Austria, 2008). We considered a two-tailed *p*-value < 0.05 to signify a significant difference between age, sex, and race groups in adjusted regression models.

## 3. Results

Table 1 shows the unadjusted distribution of the CHS sample at the baseline year, grouped by age, sex, and race, and the mean unadjusted YOL, YHL, and YAL for each group and subgroup. Most of the participants were under age 80. The subset with the smallest number of participants was non-white men age 80–100, with 50 in the group. On average, participants had 12.1 years of life, 8.3 years of healthy life, and 9.1 years of able life during 18 years of follow-up.

**Figure 1:**
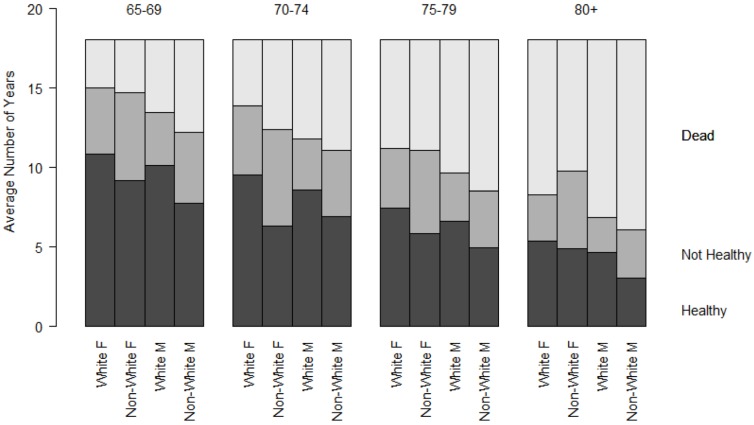
Cumulative years of healthy life (YHL) during 18 years of follow-up by age, sex, and race groups.

**Figure 2:**
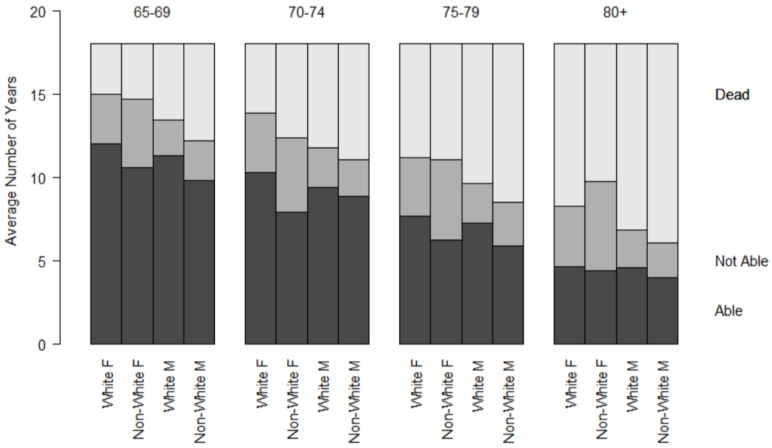
Cumulative years of able life (YAL) during 18 years of follow-up by age, sex, and race groups.

To further explore the differences noted in Table 1, Figure 1 and Figure 2 represent the cumulative YHL and YAL observed during follow-up, by age, sex, and race category. All groups total to the follow-up period, 18 years. The top section represents the amount of time lost to death, the middle section the time in the unhealthy or unable state, and the bottom section the time in the healthy or able state. There was a decline in the observed years of total, able, and healthy life with advancing age at baseline. Within groups defined by age and sex, participants across different ages spent approximately the same number of years in an unable or unhealthy state. The proportion of remaining life spent unable or unhealthy increased with advancing age categories.

**Table 1:** Distribution of the cohort at baseline, and average values of years of life, years of healthy life, and years of able life for each group and subgroup, with 95% confidence intervals shown in brackets.

Group	N (%)	% of Total	Avg YOL (95% CI)	Avg YHL (95% CI)	Avg YAL (95% CI)	% of Remaining Life Healthy	% of Remaining Life Able
Entire Cohort	5888	100%	12.1 [12.0,12.3]	8.3 [8.2,8.5]	9.1 [8.7,9.3]	69%	75%
White Females	2790 (100)	23.7%	13.1 [12.9,13.3]	9.1 [8.9,9.3]	9.8 [9.6,10.0]	69%	75%
65–69	1052 (37.7)	8.9%	15.0 [14.7,15.2]	10.9 [10.5,11.2]	12.0 [11.7,12.4]	73%	80%
70–74	861 (30.9)	7.3%	13.8 [13.5,14.2]	9.5 [9.1,9.9]	10.3 [9.9,10.6]	69%	75%
75–79	561 (20.1)	4.8%	11.2 [10.8,11.6]	7.4 [7.0,7.8[	7.7 [7.2,8.1]	66%	69%
80–100	316 (11.3)	2.7%	8.3 [7.7,8.8]	5.3 [4.9,5.8]	4.6 [4.2,5.0]	61%	53%
Non-White Females	603 (100)	5.1%	12.5 [12.1,12.9]	7.0 [6.5,7.4]	7.9 [7.5,8.4]	58%	66%
65–69	200 (33.2)	1.7%	14.7 [14.0,15.3]	9.2 [8.3,9.9]	10.6 [9.8,11.4]	63%	72%
70–74	186 (30.8)	1.6%	12.4 [11.6,13.2]	6.3 [5.6,7.0]	7.9 [7.1,8.7]	51%	64%
75–79	133 (22.1)	1.1%	11.1 [10.1,12.1]	5.8 [5.0,6.7]	6.3 [5.3,7.2]	52%	57%
80–100	84 (13.9)	0.7%	9.8 [8.7,10.8]	4.9 [4.0,5.8]	4.4 [3.6,5.2]	50%	45%
White Males	2135 (100)	18.1%	11.0 [10.8,11.3]	8.0 [7.8,8.2]	8.8 [8.5,9.0]	73%	80%
65–69	642 (30.1)	5.5%	13.5 [13.0,13.9]	10.1 [9.7,10.6]	11.3 [10.9,11.8]	75%	84%
70–74	700 (32.8)	5.9%	11.8 [11.4,12.2]	8.6 [8.2,9.0]	9.4 [9.0,9.8]	73%	80%
75–79	447 (20.9)	3.8%	9.6 [9.1,10.1]	6.6 [6.1,7.0]	7.3 [6.8,7.7]	69%	76%
80–100	346 (16.2)	2.9%	6.9 [6.3,7.3]	4.7 [4.2,5.1]	4.6 [4.2,5.0]	68%	67%
Non-WhiteMales	360 (100)	3.1%	10.4 [9.8,11.0]	6.4 [5.8,6.9]	8.1 [7.4,8.7]	62%	78%
65–69	127 (35.3)	1.1%	12.2 [11.2,13.2]	7.8 [6.8,8.7]	9.8 [8.8,10.9]	64%	80%
70–74	127 (35.3)	1.1%	11.1 [10.1,12.1]	6.9 [6.0,7.8]	8.9 [7.8,9.9]	62%	80%
75–79	56 (15.6)	0.5%	8.5 [7.1,9.9]	4.9 [3.8,6.1]	5.9 [4.6,7.2]	58%	69%
80–100	50 (13.9)	0.4%	6.1 [4.9,7.2]	3.0 [2.3,3.8]	4.0 [3.0,4.9]	49%	66%

YOL: Years of life; YHL: Years of healthy life; YAL: Years of able life.

Table 2 summarizes the regression parameters from models using YOL, YHL, and YAL as outcomes, adjusted for linear age. At the mean age of 73 years, white women had significantly greater YOL, YHL, and YAL than all the other groups, with the exception of YOL, where non-white women and white women did not differ significantly. Centered at a mean age of 73, a white female participant could expect 12.9 years of life, 8.9 of healthy life and 9.5 of able life, while a non-white female had 12.6 years of life, 7.0 years of healthy life, and 8.0 years of able life. A white male could expect 11.2 years of life, 8.1 years of healthy life, and 8.9 years of able life, and a non-white male 10.3 years of life, 6.2 years of healthy life, and 7.9 years of able life. For all three measures, white men had significantly better outcomes than non-white men. White men had significantly better YHL and YAL outcomes than non-white women, but non-white women had higher YOL than white men.

**Table 2:** Linear regressions of age and sex/race groups on YAL, YOL, or YHL for 5888 CHS participants. Coefficients represent the difference in number of years of healthy life, able life, or total life during 18 years of follow-up.

Predictor	YOL		YHL		YAL	
	Coefficient [95% CI]	*p*-value	Coefficient [95% CI]	*p*-value	Coefficient [95% CI]	*p*-value
Intercept	**12.86** [12.68,13.04]	<0.001	**8.92** [8.73,9.11]	<0.001	**9.52** [9.33,9.71]	<0.001
Baseline Age (per year)	**−0.40** [−0.42,−0.38]	<0.001	**−0.34** [−0.36,−0.31]	<0.001	**−0.43** [−0.46,−0.41]	<0.001
White Female	Reference	-	Reference	-	Reference	-
White Male	**−1.68** [−1.96,−1.40]	<0.001	**−0.81** [−1.10,−0.52]	<0.001	**−0.61** [−0.90,−0.32]	<0.001
Non-White Female	**−0.32** [−0.76,0.11]	0.14	**−1.94** [−2.39,−1.49]	<0.001	**−1.54** [−1.99,−1.08]	<0.001
Non-White Male	**−2.58** [−3.13,−2.04]	<0.001	**−2.66** [−3.22,−2.10]	<0.001	**−1.58** [−2.15,−1.02]	<0.001

YOL: Years of total life; YHL: Years of healthy life (from self-rated health); YAL: Years of able life (from no report of ADL impairments).

**Figure 3:**
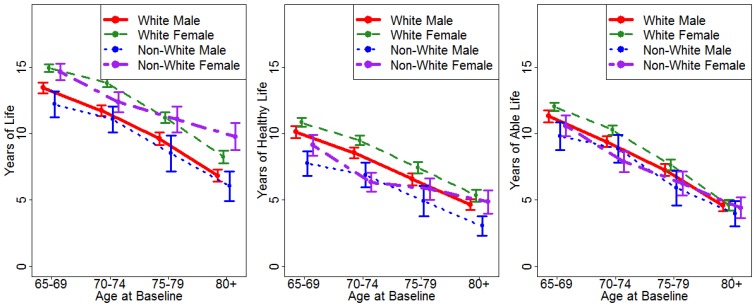
Years of life (YOL), years of healthy life (YHL), and years of able life (YAL) by age, sex, and race categories. Error bars represent the 95% confidence intervals for the point estimates.

Figure 3 shows the means of the three outcome variables, with 95% confidence intervals around the estimates. For YOL, there were significant differences between men and women at three of the four age groups (all but age 70–74), and non-white women showed a relative advantage with advancing age, so that at age 80 and over they lived the longest. Because the confidence intervals do not overlap, this is considered a significant difference. For YHL, white women showed the most years of healthy life, followed by white men, then non-white women, then non-white men. For YAL, the groups largely overlapped, especially at advanced ages.

## 4. Discussion

This is the first long-term observational study to measure years of total life, healthy life, and able life among a large cohort of older adults by using real measures over an extended time period rather than estimates from life tables, and to include a significant number of participants over age 80. In adjusted models, white women seemed to have the best outcomes, followed by white men, although our data suggest that this trend may not hold for longevity among the oldest participants. Despite a significant female advantage in terms of longevity, the sex-based differences in years of healthy and able life were not as pronounced. For these health status outcomes, race had a larger effect than did sex. These findings highlight differences in health status by sex, age, and race, which may not be recognized if only survival is examined, or if samples are too small to stratify by age groups.

The differences between race and sex groups seen in the figures and the adjusted regression models were substantial, with a difference of more than two and one-half years of healthy life for white women compared to non-white men. In Figure 1 and Figure 2, this is seen by the difference in the lower shaded bars in groups of the same age. This represents more than just an academic difference, since it translates into years of reduction in health and capacity, and would, by inference, diminish the cumulative well-being and independence of individuals in different groups.

It is noteworthy that the amount of time spent in unable or unhealthy states (the lightly-shaded areas in Figure 1 and Figure 2) are about the same across age groups for each race and sex category, indicating that the reductions in lifespan come out of potentially able or healthy years. Put another way, having a shorter lifespan did not result in fewer *total years* in an unable or sick state. Conversely, those with a longer lifespan after baseline did not have fewer years unable. As a consequence, the *proportion* of remaining life spent in an unhealthy or unable state increased with increasing age categories. This observation, observed previously using transition probabilities [20], could help to direct future research about racial and ethnic disparities in older age, by examining more nuanced patterns besides longevity.

The differences between race and sex groups generally held across all age ranges, except that YOL diverged more at the oldest age group, while YAL converged. The relative differences in YHL did not change much by age. As with the differences in total, healthy, and able life years, these findings discourage making general inferences from any of the domains to the others. In this way, our results differ from previous investigations based on life tables which assume that the prevalence of healthy and able states is homogeneous over time, and also from research which applies homogeneous transition probabilities in Markov models (see a recent study about trends in mortality for more detail about the limitations of multistate life tables [21]).

One unexpected observation in this analysis was that non-white women in the oldest age group at baseline had significantly more years of total life, as seen in Figure 3. This may be a result of the relatively small sample, with 84 non-white women starting the CHS study in the over age 80 group. There may have been a selection bias in that octogenarians who chose to participate in research may have been particularly healthy, and such selection bias may have been more pronounced in socially disadvantaged sub-groups. Nonetheless, this may suggest a particular survival advantage for older non-white women, albeit with relatively more time spent unable or unhealthy.

This research was limited, as other studies were, by sample selection. Cohort 1 of the CHS study, for instance, was almost exclusively white, and Cohort 2 included only black participants, who were recruited three years later. In order to have groups large enough to analyze, we dichotomized race as white or non-white, and there may be other racial and ethnic differences that we could not identify. Because the cohort came from only four study sites, we were not able to investigate the effects of geographical location, which in previous research has shown significant effects on mortality, and interactions with race [11]. All the health and functional outcomes were self-reported, and were not compared to objective health measures. There may be differences in reporting of disability or illness in different age and sex groups that create the impression of better or worse outcomes in health and activity, although we maintain that regardless of objective health status, self-reported functional impairments and fair or poor health constitute intrinsically negative states for the individuals who report them. We defined ability by the performance of six ADLs, which may underestimate the global independence of some participants, although other research has found that altering the threshold for disability has little effect on able life expectancy estimates [22]. Five out of 18 years of activity data were missing and had to be imputed. Additional data was filled in by linear interpolation. We used only person-level imputation, and excluding all these years in a sensitivity analysis did not modify the differences observed between groups (results are not shown). There were relatively few participants at very advanced ages, but the confidence intervals, seen in Figure 3, were small enough to allow meaningful comparisons. In order to examine age, race, and sex trends in detail, we did not control for other sociodemographic, health-related, clinical, or behavioral factors, which may contribute to mortality [23,24,25], and it is possible that these confound the observed differences between groups.

## 5. Conclusions

Using long-term observations in a large cohort of American older adults, we found significant racial and gender-based differences in years of total life, years of healthy life, and years of able life. Gender had a larger effect on longevity than did race, but race had a larger effect on years spent healthy or able than did gender. The sociodemographic, health utilization, and behavioral factors that may be implicated in these differences merit further attention.
